# Investigation of the out-of-plane load-bearing capacity of T1100/5405 composite T-stiffened panels

**DOI:** 10.1038/s41598-021-00275-w

**Published:** 2021-10-22

**Authors:** Heyuan Huang, Xuanjia Zhang, Zhicheng Dong, Dong Wang

**Affiliations:** 1 School of Aeronautics, Northwestern Polytechnical University, Xi’an, 710072 China; 2 Aircraft Strength Research Institute, Aviation Industries of China, Xi’an, 710072 China

**Keywords:** Aerospace engineering, Composites

## Abstract

With the continuous improvement of the mechanical properties of composite materials, the adhesive interface performance of composite T-stiffened panels has become a critical factor in determining the overall structural strength. However, little work has been reported on the mechanical properties of adhesive interfaces in composite T-stiffened panels under lateral bending and shear loading. Especially, there is no clear explanation on the damage evolution law of structural properties for the interface with defects, which greatly influenced the use of T-stiffened composite structures. In this paper, the mechanical properties of T1100/5405 composite T-stiffened laminates under lateral bending and shear loading are experimentally and numerically investigated. The load-bearing capacities for the panels with intact and defected adhesive interfaces are compared, the damage evolution law of typical T-stiffened structures is further explored. Based on the continuum damage model (CDM) and the cohesive zone model (CZM), the constitutive models of the adhesive layer and the composite material are established respectively. Good agreements between experimental and numerical profiles illustrate that damages mainly occur on the loading side and the corner of the L-type ribs under lateral bending conditions, while damages extend from both sides of the interface layer to the center under shear loading. When a prefabricated defect exists, damages extend from the defect location along the loading direction. At the same time, the analysis shows that the lay-up of the surface layer, the chamfer radius, and the width of T-type ribs have a great influence on the structural load-bearing capacity, but less on the damage evolution form.

## Introduction

As a common structure form, composite stiffened panels are mainly used in the main load-bearing structure of aircraft and rockets and are widely used in the design of aircraft structures^1–5^. The most common stiffened panels are T-type, L-type, J-type, π-type, etc.^6,7^. Compared with the J-type structure, which has a complex configuration and can only transmit special loads, T-stiffened panels with manufacturing process advantages are more widely used in canards, wings, fuselages, and other parts of aircrafts^8^.

At present, a lot of research has been done on T-stiffened panels domestically and abroad. The most common research is on mechanical properties and failure mechanisms under tensile loading. Bai et al.^9^ obtained the load–displacement and damage evolution behavior of T-stiffened panels through tensile tests, established the progressive damage model based on Hashin, Chang-Chang, Hou, and hybrid criteria, and discussed the influence of the radius of triangle on the failure mode. Luo et al.^10^ tested the tensile mechanical properties of T-joint through experiments and studied the failure process. Wu et al.^11^ analyzed the progressive damage mode of T-joint under tensile loading from the perspective of numerical simulation. In terms of structural buckling, Ye et al.^12^ studied the interfacial properties of skin and stiffened panels and the effects of layering on debonding and revealed the effects of different bonding methods on the buckling failure modes of composite T-stiffened panels after compression. In the study of stiffened panels with defects, Ji and Zhao et al.^13^ studied the debonding behavior with defects, adopted a progressive damage model to predict the expansion of interlaminar damage, and used a bilinear cohesion model to evaluate the debonding damage between skin and stiffener web, and the damage evolution law of this model was in good agreement with the experimental situation. In terms of damage location, Bai et al.^14^ explored the influence of different damage locations on the bearing capacity and failure mode of the stiffened panels by comparative analysis of the bearing capacity and failure mode of the ribs, skin, and interface bonding layer with elliptical damage under a compression load, as well as the verification of the finite element model. Jasto and Reinoso et al.^15^ conducted experimental analysis on the initial position and propagation path of T-stiffened panels damage and concluded that the damage started from the triangular position of the T-joint and expanded asymmetrically to both sides, which was consistent with the conclusion drawn by Zhu^16^ and Cheng et al.^17^. Li et al.^18^ studied the damage propagation and failure characteristics of composite stiffened panels with notches under compression loading by experimental and finite element methods. They also further analyzed the effect of notches on the residual strength of the panels and predicted the failure mode by combining the maximum stress criterion and Hashin criterion. Meeks^19^ explored the failure mechanism of skin-stiffeners debonding in five post-buckling panels of Greenhalgh^20^ and believed that the main failure mode of these panels was the debonding of stiffeners.

It can be concluded from the above research that the bonding interface is the weak part of the T-stiffened panels, especially defects such as pores, inclusions, poor glue, and poor fiber/matrix interface will occur in the manufacturing or service process of composite materials^21–23^. Damaging structures will reduce the load-bearing capacity of structures to a certain extent^20,24^, especially for super-strong composite materials such as T1140/5405, the interface performance is the key factor that determines the structural performance. Therefore, it is necessary to study the failure of damaged T-stiffened panels. However, most of the current studies mainly focus on the bearing capacity and damage mechanism of adhesive joints under tensile and compression loads, as well as the strength and delamination of the panel itself^25–27^. In the actual load-loading process of aircraft, T-stiffened panels also transmit various complex loads, such as lateral bending and in-plane shear, etc. At present, the research on this aspect is relatively scarce, so this paper starts research on this basis.

In this paper, we respectively studied the structural load-bearing capacity and interfacial damage evolution law of composite T-stiffened panels with intact and defected structure under lateral bending and shear loads. First, four types of T-stiffened panels with intact and defected structure were designed and fabricated. The basic mechanical performance parameters of T1100/5405 and the mechanical performance data of the structural parts under lateral bending and shear loads were obtained through experiments. Then, based on the continuum damage mechanics model (CDM) and the cohesive zone model (CZM), a progressive damage model is established. The load-bearing capacity and damage propagation law of composite T-stiffened panels with intact and defected structure were compared and analyzed from two aspects of test and finite element simulation, so as to effectively predict the strength and damage evolution of T-stiffened panels. Finally, based on the numerical model, the influence of design parameters on the bonding interface performance of T- stiffened panels are analyzed, and the structural optimization design scheme is given accordingly.

## Experiment

### Basic mechanical properties test

**Figure 1:**
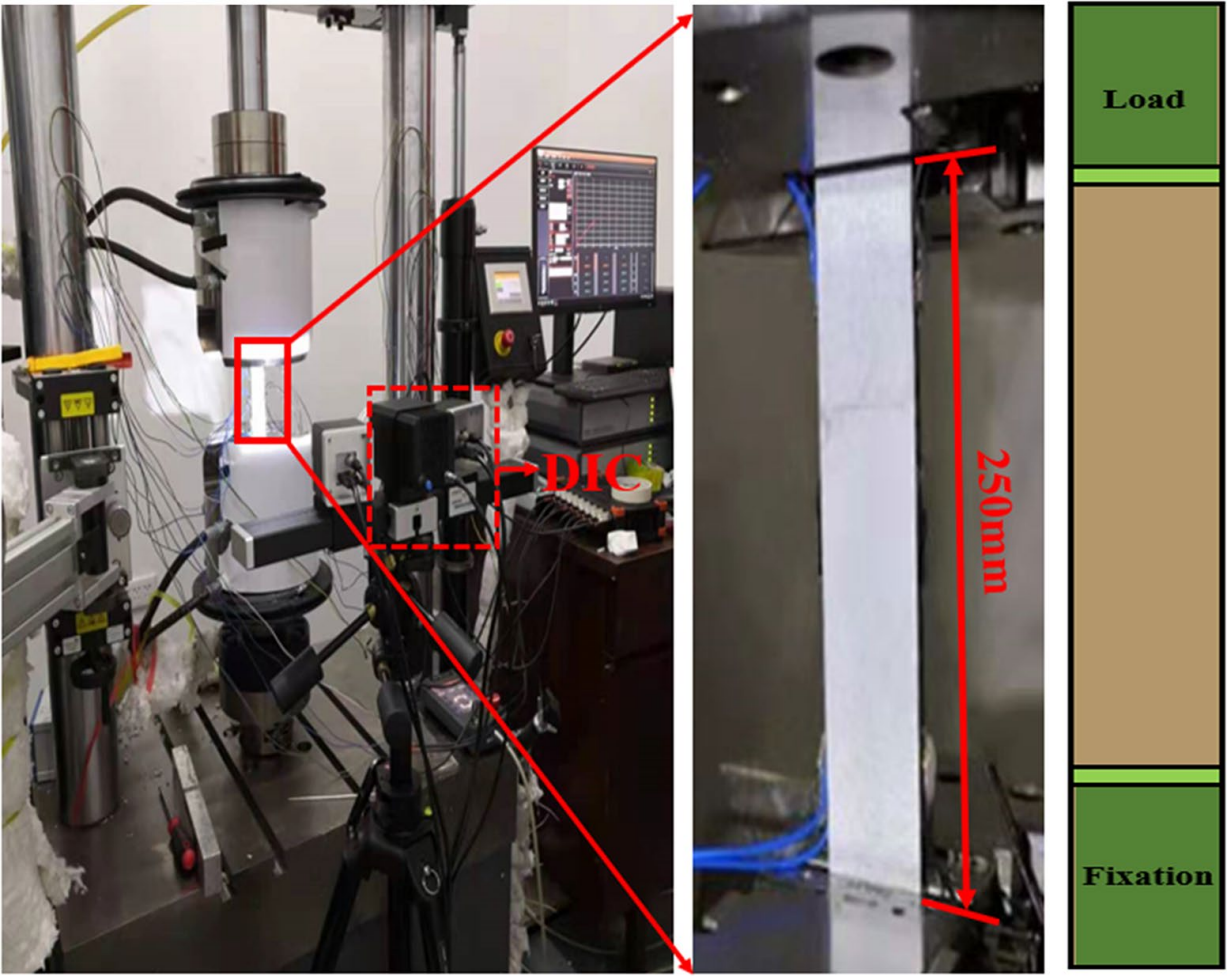
Loading form and testing device for basic mechanical properties of laminates.

**Figure 2:**
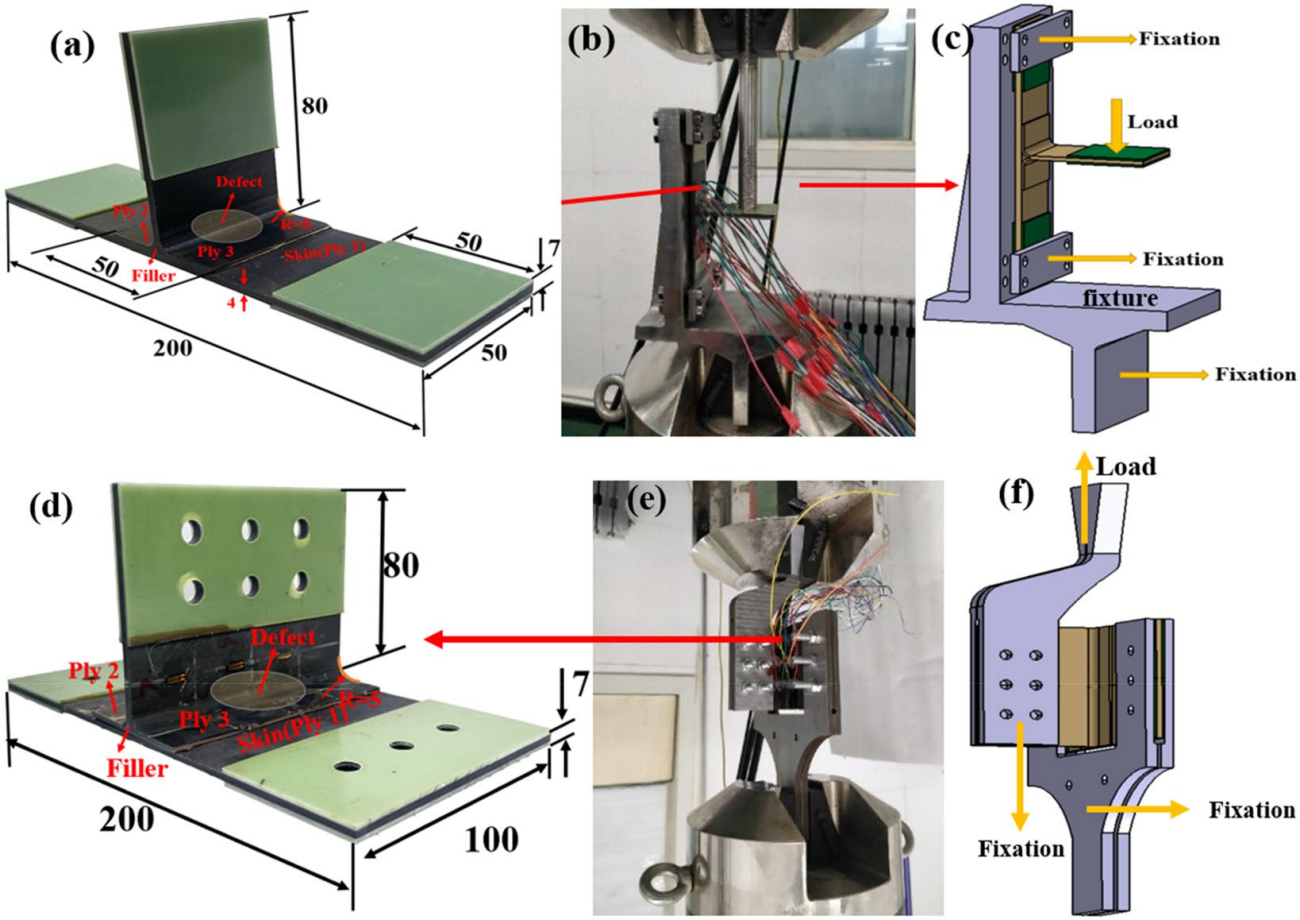
Schematic diagram of T-stiffened panel structure test piece and loading device: (**a**) T-lateral bend-stiffened panel dimension; (**b**) side bending test loading device; (**c**) lateral bend test geometry; (**d**) T-shear-stiffened panel dimension; (**e**) shear test loading device; (**f**) shear test geometry.

Basic mechanical property tests of T1100/5405 composite laminates were carried out to obtain the basic mechanical property parameters of the materials and provide data support for subsequent analysis. The test matrix is shown in Table 1. The test device is shown in Fig. 1. Switzerland w + b universal testing machine is used as loading equipment. 0^o^ and 90° compression test using anti-instability fixture, both ends of the test piece through the testing machine chuck fixed. The combination of a static strain test analysis system and Digital Image Correlation (DIC) is adopted to collect the strain data of the test area. There are 6 samples of each type. The specimen is a standard specimen, and the size of one specimen is given in Fig. 2. The average value of test results is shown in Table 2. Table 2 compares the basic properties of two common composite materials at the same time. It can be concluded that the performance of T1100 is greatly improved compared with that of T700 and T300.

**Table 1:** Test matrix for basic mechanical properties of composite laminates.

Test project	Layer	Test standard	Quantity	Test content
0° tensile test	[0]_8_	ASTM D3039^28^	6	$X_{ft} ,E_{ft}$
0° compression test	[0]_16_	ASTM D6641^29^	6	$X_{fc} ,E_{fc}$
90° tensile test	[90]_16_	ASTM D3039^28^	6	$X_{mt} ,E_{mt}$
90° compression test	[90]_16_	ASTM D6641^29^	6	$X_{mc} ,E_{mc}$
Interfacial shear	[± 45]_5s_	ASTM D3518^30^	6	$G_{{{12}}} ,S_{{{12}}}$

*X*_ft_, *X*_*fc*_, *X*_*mt*_, *X*_*mc*_, and *S*_*12*_ are respectively longitudinal tensile strength, longitudinal compression strength, transverse tensile strength, transverse compression strength, and in-plane shear strength. *E*_*ft*_*, E*_*fc*_*, E*_*mt*_*, E*_*mc*_, and *G*_*12*_ are respectively longitudinal tensile modulus, longitudinal compression modulus, transverse tensile modulus, transverse compression modulus, and in-plane shear modulus.

**Table 2:** Mechanical properties of T1100/5405 and common carbon fiber composites.

Test results of basic mechanical properties of T1100/5405 composites					
	*X*_*ft*_ (MPa)	*X*_*mt*_ (MPa)	*X*_*fc*_ (MPa)	*X*_*mc*_ (MPa)	*S*_*12*_ (MPa)
T1100/5405	2733.5	61.57	1470.22	216.312	95.275
Standard deviation	182.42	5.66	151.84	18.2	2.3

	*E*_*ft*_ (GPa)	*E*_*mt*_ (GPa)	*E*_*fc*_ (GPa)	*E*_*mc*_ (GPa)	*G*_*12*_ (GPa)
T1100/5405	324	8.9	176.501	14.227	5.658
Standard deviation	6.68	0.47	14.41	19.57	0.245

Mechanical properties of common composite materials					
	*X*_*ft*_ (MPa)	*X*_*mt*_ (MPa)	*X*_*fc*_ (MPa)	*X*_*mc*_ (MPa)	*S*_*12*_ (MPa)
T700/QY8911^11–13^	1830	31.31	895	125	71.97
T300/QY8911^14,25,31^	1548	55.5	1226	110.5	89.9

	*E*_*ft*_ (GPa)	*E*_*mt*_ (GPa)	*E*_*fc*_ (GPa)	*E*_*mc*_ (GPa)	*G*_*12*_ (GPa)
T700/QY8911^11–13^	230	7.4	97.9	7.05	4.09
T300/QY8911^14,25,31^	230	8.8	85	8.2	4.47

### T-type panels test

The webs of the T-stiffened panels are composed of two L-type ribs (marked as ply 2 and 3) and the ply is bonded with the skin by J116B. The dimensions are shown in Fig. 2. The lay-up sequence of skin is [45/0/− 45/90/0/90/− 45/0/45/0]s, and the lay-up sequence of T-stiffened panels ply 2 is [45/− 45/0/45/45/− 45/0/45/− 45/90/45/− 45], ply 3 arranged in a [− 45/45/90/− 45/45/0/− 45/45/45/0/− 45/45]. There are six pieces of each specimen. The longitudinal dimension (L = 100 mm) of the T-stiffened panels’ specimen in the shear test is twice that of the side bend specimen (L = 50 mm), and the dimensions, layup, and other parameters are the same as the side bend test piece. A foil with a radius of 15 mm is embedded between the L-type ribs and the skin layer to simulate the defects of inclusion, poor glue, and poor fiber/matrix interface in the manufacturing process of t1000/5045 carbon fiber composites, as shown in Fig. 2a,d. T structure specimen uses PLD-250 universal testing machine and its load accuracy is ± 1%. In the T-bending experiment, the six degrees of freedom at both ends of the bottom surface of the T-shaped sample were fixed with a clamp. The chuck on the test machine exerts downward lateral bending force on the T-shaped plate and the loading diagram is shown in Fig. 2c. In the T-type shear test, the six degrees of freedom at both ends of the bottom of the T-type specimen are fixed with a clamp. According to the characteristics of the fixture, the chuck on the test machine is controlled to move upward by 2 mm/min to achieve shear loading. The schematic diagram of loading is shown in Fig. 2f. During the test, the fixtures shown in Fig. 2c,f were independently designed and manufactured to implement the lateral bending load and shear load. The test results are shown in Table 3. The dispersion coefficient of the specimen results is within the acceptable range. Compared with the intact structure, under the lateral bending load, the failure load of the defected T-stiffened panels is reduced by 15.38%, and the shear ultimate strength of the T-stiffened panels is reduced by 7.26%.

**Table 3:** Summary of test results.

	Non-defective lateral bend (kN)	Defective lateral bend (kN)	No-defective shear (MPa)	Defective shear (MPa)
Failure load/strength	0.689	0.583	5.768	5.349
Standard deviation	0.069	0.066	0.213	0.483
Dispersion coefficient	10.01%	11.32%	3.69%	9.01%

### Numerical simulation method

#### Material constitutive model

##### CZM

**Figure 3:**
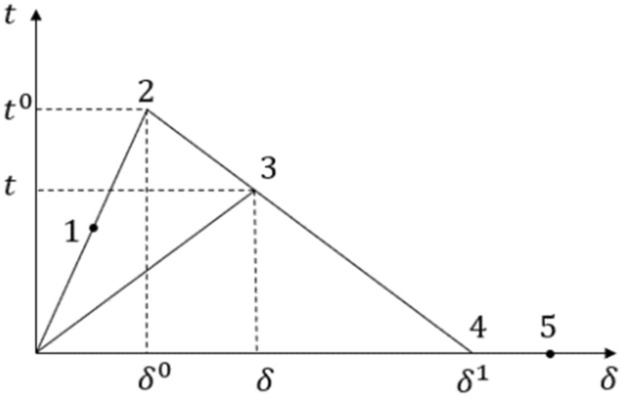
Bilinear constitutive model.

A layer of cohesive units is set between the ribs and the skin, and the bilinear constitutive model shown in Fig. 3 is used to simulate the mechanical behavior of the interface glue layer. When the interface is intact, the stress increases linearly with the increase of the displacement of the cohesive unit (the line segment 1–2 in Fig. 3). At point 2 in Fig. 3, the element stress of the interface layer reaches its limit and the stiffness begins to decay. After the interface damage starts, the interface material performance undergoes stiffness degradation according to the bilinear constitutive model to actualize the simulation of the damage initiation and damage evolution of the interface layer^32–34^.

The expression of the bilinear constitutive model is as follows:

1. When $\delta < \delta^{0}$, as linear elastic interface layer, constitutive relationship is
  1 $$ t = K\delta $$
  where K is the stiffness matrix of the cohesive force element.
2. When $\delta^{0} < \delta < \delta^{1}$, constitutive relationship is
  2 $$ t = (1 - d)K\delta $$
  where d is the damage accumulation factor. When d = 1, the interface layer is completely destroyed.
3. When $\delta > \delta^{1}$, The interface stiffness degrades to zero and expands in layers.

Use the secondary nominal stress^35–37^ in the traction–separation rule to define the initial damage:

3 $$ \left\{ {\frac{{\left\langle {t_{n} } \right\rangle }}{{t_{n}^{0} }}} \right\}^{2} + \left\{ {\frac{{t_{s} }}{{t_{s}^{0} }}} \right\}^{2} + \left\{ {\frac{{t_{t} }}{{t_{t}^{0} }}} \right\}^{2} = 1 $$

where $t_{n}^{0}$ is the normal tensile strength of the cohesive element; $t_{s}^{0} ,t_{t}^{0}$ is the two shear strengths perpendicular to the crack surface; $\left\langle t \right\rangle = (t + \left| t \right|)/2$ means assuming that the compression displacement does not affect the failure of the element.

In predicting the stratified expansion, the damage expansion is defined based on the energy method. When the interlayer energy release rate is greater than the critical energy release rate, the delamination expands. In the layered expansion criterion of laminates, the B-K^38,39^ criterion is adopted:

4 $$ G_{{\text{T}}}^{m} = G_{Ic} + (G_{IIc} - G_{Ic} )\left( {\frac{{G_{II}^{m} }}{{G_{I}^{m} + G_{II}^{m} }}} \right)^{c} $$

where $G_{Ic} ,G_{IIc} ,G_{T}$ is type I, type II and the total critical energy release rate, and c is the mixed mode parameter.

##### CDM

Based on the continuum damage mechanics model, a composite material constitutive model is established for the analysis of the damage evolution of ribs and skins. The damage variable of the material is expressed in the form of a tensor, and the constitutive relationship of the composite material can be expressed as the following form:

5 $$ \sigma = C_{D} \varepsilon $$

where $\sigma ,\varepsilon ,C_{D}$ is stress, strain and matrix of stiffness under the principal axis of material damage^40^.

Three-dimensional Hashin^41,42^ failure criterion is adopted for material failure determination, as shown in Table 4:

**Table 4:** Hashin failure criteria.

Fiber tensile failure	$\left( {\frac{{\sigma_{11} }}{{X_{T} }}} \right)^{2} + \left( {\frac{{\tau_{12} }}{{S_{12} }}} \right)^{2} + \left( {\frac{{\tau_{13} }}{{S_{13} }}} \right)^{2} \ge 1$
Fiber compression failure	$\left( {\frac{{\sigma_{11} }}{{X_{c} }}} \right)^{2} \ge 1$
Matrix tensile failure	$\left( {\frac{{\sigma_{22} }}{{Y_{T} }}} \right)^{2} + \left( {\frac{{\tau_{12} }}{{S_{12} }}} \right)^{2} + \left( {\frac{{\tau_{23} }}{{S_{23} }}} \right)^{2} \ge 1$
Matrix compression failure	$\left( {\frac{{\sigma_{22} }}{{Y_{c} }}} \right)^{2} + \left( {\frac{{\tau_{12} }}{{S_{12} }}} \right)^{2} + \left( {\frac{{\tau_{23} }}{{S_{23} }}} \right)^{2} \ge 1$
Fiber-matrix shear failure	$\left( {\frac{{\sigma_{11} }}{{X_{c} }}} \right)^{2} + \left( {\frac{{\tau_{12} }}{{S_{12} }}} \right)^{2} + \left( {\frac{{\tau_{13} }}{{S_{13} }}} \right)^{2} \ge 1$

Where $\sigma_{11} ,\sigma_{22}$ is the normal stress and $\tau_{12} ,\tau_{13} ,\tau_{23}$ is the shear stress in three directions respectively; $X_{T} ,X_{C}$ is the longitudinal tensile and compressive strength, $Y_{T} ,Y_{C}$ is the transverse tensile and compressive strength, and $S_{ij}$ is the in-plane shear strength.

##### Finite element model

**Figure 4:**
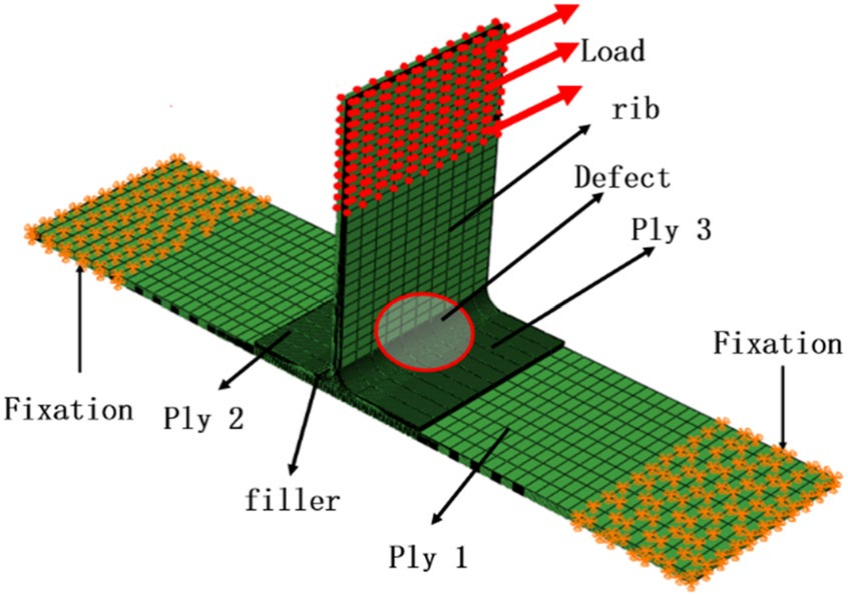
(Lateral bending & Shear) Finite element model (ABAQUS 6.14, https://www.3ds.com/products-services/simulia/products/abaqus)^43^.

ABAQUS 6.14 software is adopted to establish the three-dimensional finite element model of T-stiffened panels, as shown in Fig. 4. The mechanical performance parameters of the cohesive layer are shown in Table 5. The structure consists of ply 1, ply 2, ply 3, and fillers. A layer of the cohesive unit is established on the contact surface of Ply 1 and Ply 2, 3 and filler to simulate bonding, and the filler is in cohesive contact with Ply 2 and 3. For the T-type model with damage, delete the cohesive element at the corresponding position of the defects. In the finite element analysis, both ends of the bottom of the specimen (yellow part in Fig. 4) were fixed with six degrees of freedom. Displacement load was applied on the top of the rib (red part in Fig. 4) and the direction was parallel to the bottom surface and the plane of the rib (red arrow in Fig. 4). The finite element boundary conditions of lateral bending and shearing are consistent with the experimental boundary conditions.

**Table 5:** Mechanical properties of cohesive element^17,44^.

G_*Ic*_ (kJ/m^2^)	G_*IIc*_ (kJ/m^2^)	${t}_{n}^{0}$ (MPa)	${t}_{s}^{0}$ (MPa)	${t}_{t}^{0}$ (MPa)
0.111	0.501	13.474	6.999	6.999

## Results and discussion

### Model validation

**Figure 5:**
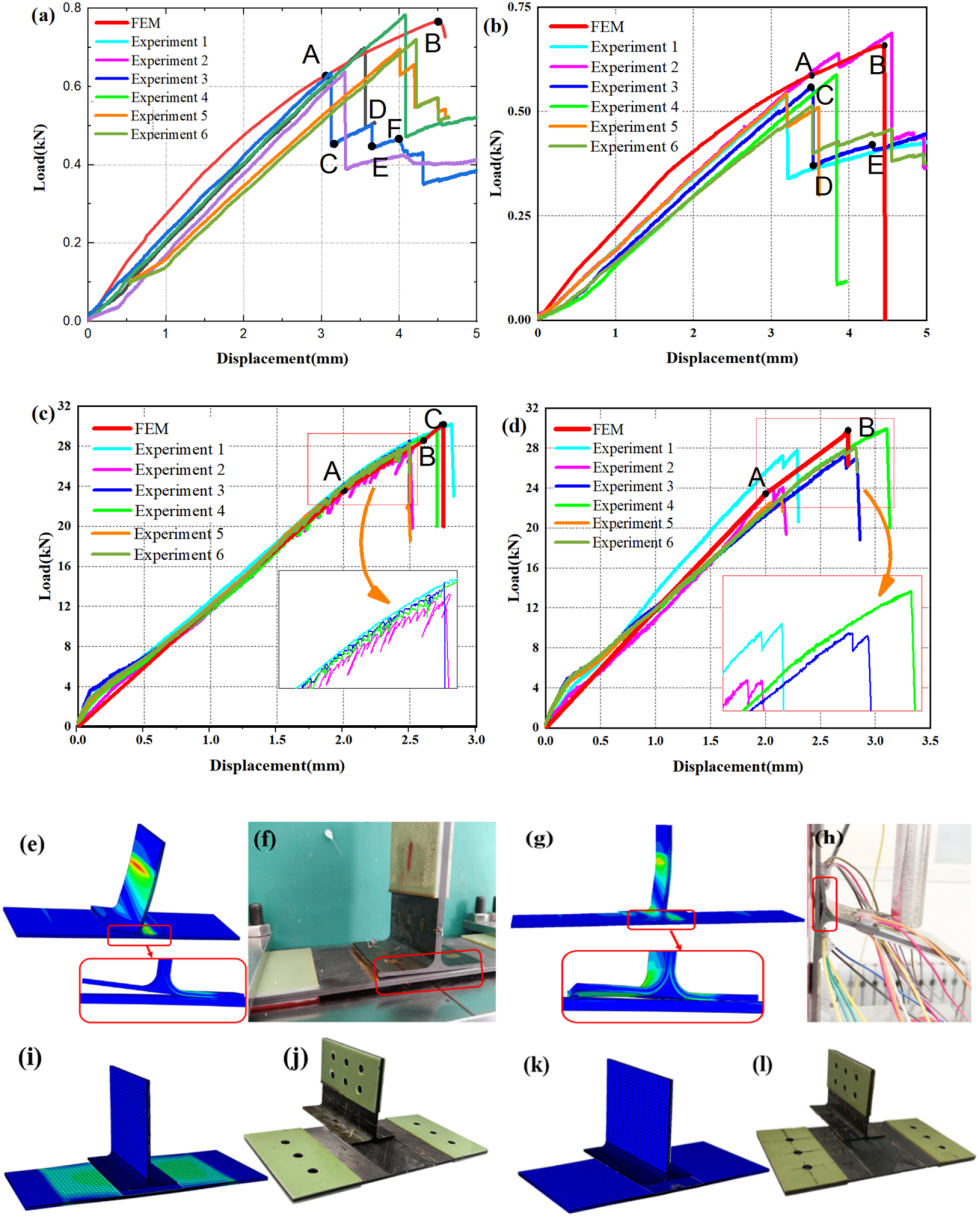
Comparison of test and finite element results: (**a**) Non-defective side bending; (**b**) side bending with defects; (**c**) non-defective shearing; (**d**) shear with defects; (**e**,**f**) Non-defective side bending deformed structure and model; (**g**)–(**l**) Defective side bending deformed structure and model; (**i**,**j**) Non-defective shearing deformed structure and model; (**k**,**l**) Defective shear deformed structure and model (ABAQUS 6.14, https://www.3ds.com/products-services/simulia/products/abaqus)^43^.

Figure 5 is a comparison diagram of load–displacement curves obtained from experiment and numerical simulation. The results of finite element and experimental tests are presented in Table 6. It can be seen from Fig. 5 and Table 6. that for the lateral bend, the test result of the intact T-stiffened panels is 0.689 kN, the finite element calculation result is 0.766 kN, and the calculation error is 11.17%; the test limit load of the damaged structure is 0.583 kN, the limit load calculated by the finite element is 0.657 kN, and the calculation error is 12.69%. For T-shear, the failure load obtained by the intact structure experiment is 28.83 kN, the failure load calculated by the finite element method is 30.190 kN, and the calculation error is 4.72%; the failure load obtained by the damaged structure experiment is 26.84 kN, and the failure load calculated by the finite element method is 29.795 kN, and the calculation error is 11%. The above results show that the numerical simulation has high accuracy and can meet the requirements of subsequent analysis. The comparison also shows that the lateral bending bearing capacity is much lower than the shear bearing capacity. This is mainly caused by two reasons: one is that the lateral bending load is transmitted to the skin through the web, and the load is transformed into tension on one side at the interface layer, and the other side is compressed, and the compressed side bears most of the load. The damage mainly occurs on the side under tension and at the corners of T-stiffened panels. The other is that the stress concentration in the triangle area is more serious than under the shear load because of the bending moment. The second is that under shear load, the stress distribution at the bonding interface is uniform, and the entire interface is loaded. In addition, the width of the ribs is also one of the important factors affecting the load-bearing performance of the structure.

**Table 6:** Results of finite element and experimental tests.

	Lateral bending load		Shear load	
	Intact specimen	Defective specimen	Intact specimen	Defective specimen
Experiments	0.689 kN	0.583 kN	28.83 kN	26.84 kN
FEM	0.766 kN	0.657 kN	30.19 kN	29.795 kN
Error (%)	11.17	12.69	4.72	11

In the lateral bending test, as the load increases before structural damage, the load–displacement relationship of the OA section is linear, and the curve appears stepped after loading to the failure load (as shown in Fig. 5a CD, EF segment, and Fig. 5b DE segment). This is because after cracks appear at the interface, the structure still has a certain bearing capacity, and as the displacement increases, the load increases slightly, the cracks continue to expand, and the structural rigidity decreases. After the structure fails, fiber bridging occurs at the crack propagation interface. For the lateral bending specimens without prefabricated defects, taking experiment 3 as an example, when the load reaches 0.62 kN, cracks appear, corresponding to the initial damage at point A in Fig. 5a; the corresponding load with prefabricated defects is slightly lower. In the process of loading the lateral bending, the skin is slightly bent to the side of the rib due to its low stiffness, so the curve does not completely rise linearly before reaching the limit load (as shown in Fig. 5a,b OA segment).

In the shear test, when the load reached about 23 kN, initial damage occurred and a small amount of fiber breaking sound was heard. In the finite element simulation, corresponding to point A in Fig. 5c, the stiffness of section AB decreased compared with that before the damage. Figure 5c shows that when the specimens with no prefabricated defects are close to failure, the curve has several non-linear minor disturbances, which are caused by the decrease of the rigidity of the laminate after the fiber damage occurs. Because of the precast defects, the shear load in Fig. 5d decreases slightly when it reaches the critical value and then increases. When the shear load reaches the limit, the bonding interface between the rib and the skin is almost instantaneously debonded, and a small amount of fiber on the surface of the interface is pulled out and broken.

Figure 5e–l show the deformation structure of the finite element and test. In a side-bending test, the ribs gradually tilt as the load increases, and the layers appear initially in the triangle and eventually completely separate. The finite element results of adhesive cracking are consistent with the test, as shown in Fig. 5g,h. But the bending deformation part of the ribs is not the same, which is because the load in the finite element is applied on the whole loading end of the rib. However, considering the higher stiffness of the actual test specimen rib, the bending load in the test is applied in the middle of the loaded end. As shown in Fig. 5i–l, rib and cortex were completely separated in shear test and finite element simulation.

### Failure analysis

#### Lateral bending load

**Figure 6:**
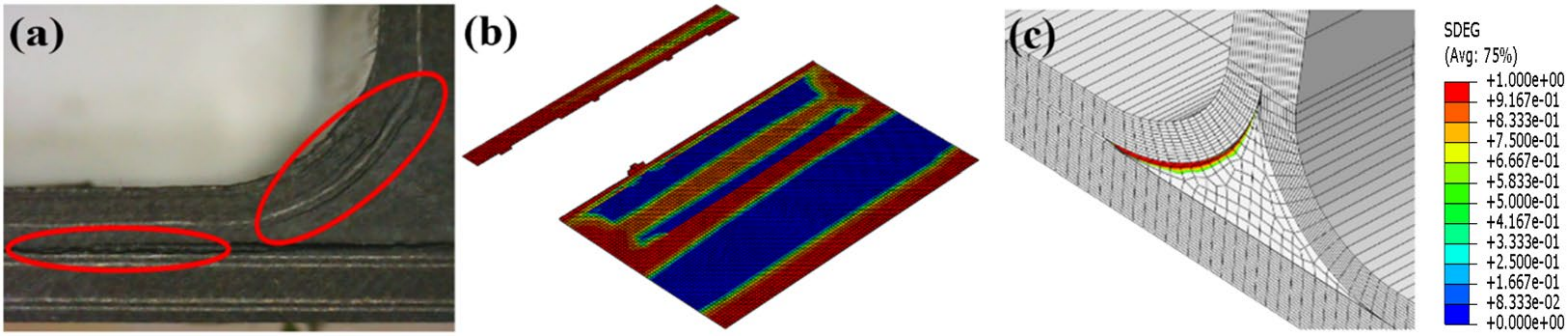
Failure modes of side bending interface without prefabrication defect: (**a**) Test result; (**b**) numerical result of adhesive layer; (**c**) numerical result of filler (ABAQUS 6.14, https://www.3ds.com/products-services/simulia/products/abaqus)^43^.

**Figure 7:**
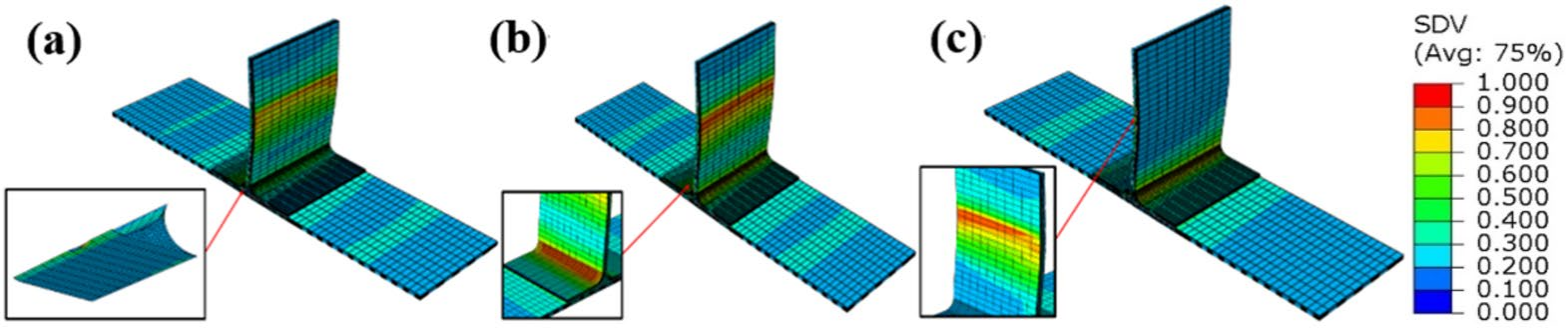
Failure modes of side bending materials without prefabrication defects: (**a**) fiber failure; (**b**) matrix failure; (**c**) fiber-matrix shear failure (ABAQUS 6.14, https://www.3ds.com/products-services/simulia/products/abaqus)^43^.

Figure 6 is a diagram showing the damage mode of the intact structure under side bending load, and Fig. 7 is a schematic diagram of the failure mode of structure without prefabrication defects. The failure of T-stiffened panels under lateral bending load (Fig. 6a) mainly occurs on the loaded side (Fig. 6b) and the corner of L-type ribs (Fig. 6c), and the stress level in the triangle area is the highest. Cracks first appear in the rounded area on the loaded side and at the bonding interface between the bottom corner of the triangular filling area and the skin. When the structure is damaged, its local stiffness decreases. Stress and force transmission routes throughout the structure will be redistributed. As the load increases, the degree of delamination or stripping caused by the initial damage will gradually increase. Several cracks will appear in the joint structure, and the initiation of cracks is mostly random microcracks. The cracks in the rounded area extend upward along the L-type ribs, and the cracks in the triangular area extend to both sides. The expansion speed of cracks to the left is faster, and there is still a part of the interface at the leftmost end of the loaded side that is not completely debonding, and its failure mode is a mixed type I and type II failure. The degumming behavior is accompanied by fiber bridging and fiber fracture. As shown in Fig. 7, partial shear damage occurs on one side of the fiber and matrix under load at the loading end, and partial compression damage occurs on the other side of the fiber and matrix. At the corner of L-type ribs, partial tensile damage occurs on the matrix due to stress concentration. In the web plane of the ribs, one side of the load is tensile strain, and the other side is compressive strain. The skin strain is relatively small relative to the ribs as a whole.

**Figure 8:**
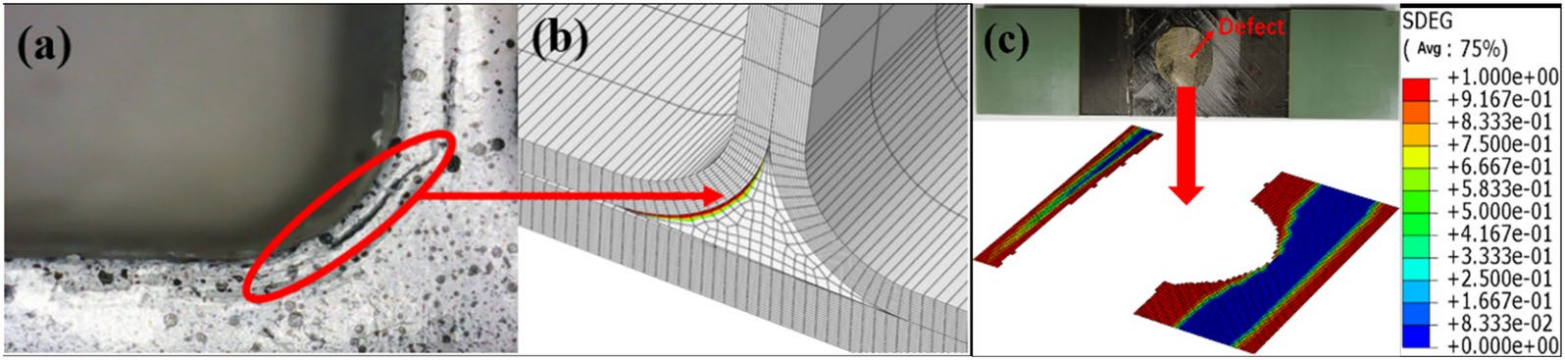
Failure modes of side bending interface with defect: (**a**) failure mode of test with defect; (**b**) interface layer failure 1; (**c**) interface layer failure 2 (ABAQUS 6.14, https://www.3ds.com/products-services/simulia/products/abaqus)^43^.

**Figure 9:**
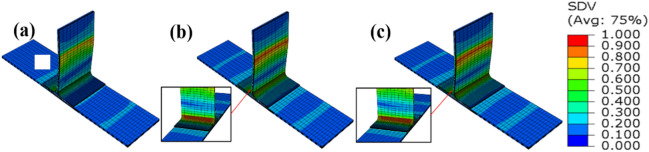
Failure modes of side bending material with defect: (**a**) fiber failure; (**b**) matrix failure; (**c**) fiber-matrix shear failure (ABAQUS 6.14, https://www.3ds.com/products-services/simulia/products/abaqus)^43^.

The simulation results of the side bending damage evolution of the T-stiffened structure with prefabricated defects are shown in Figs. 8 and 9 The load is transmitted to the skin through the rib web, and the load direction changes in the triangular filling area. According to the test results of Fig. 8a, a small number of damage cracks appeared at the defect location first, and the cracks extended downward along the arc path, and further extended to the left as the load increased. The damage propagation form was consistent with the simulation results. Secondly, due to the existence of prefabricated defects in the cementing interface of the T-joint, the simulation results show that the damage extends from the circular defect to both sides, and then extends to the loading direction until it is completely debonding, as shown in Fig. 8c. Since the prefabricated defect is located in the interface layer, the failure mode of the fiber and matrix in Fig. 9 is not much different from that without prefabricated defects.

#### Shear load

**Figure 10:**
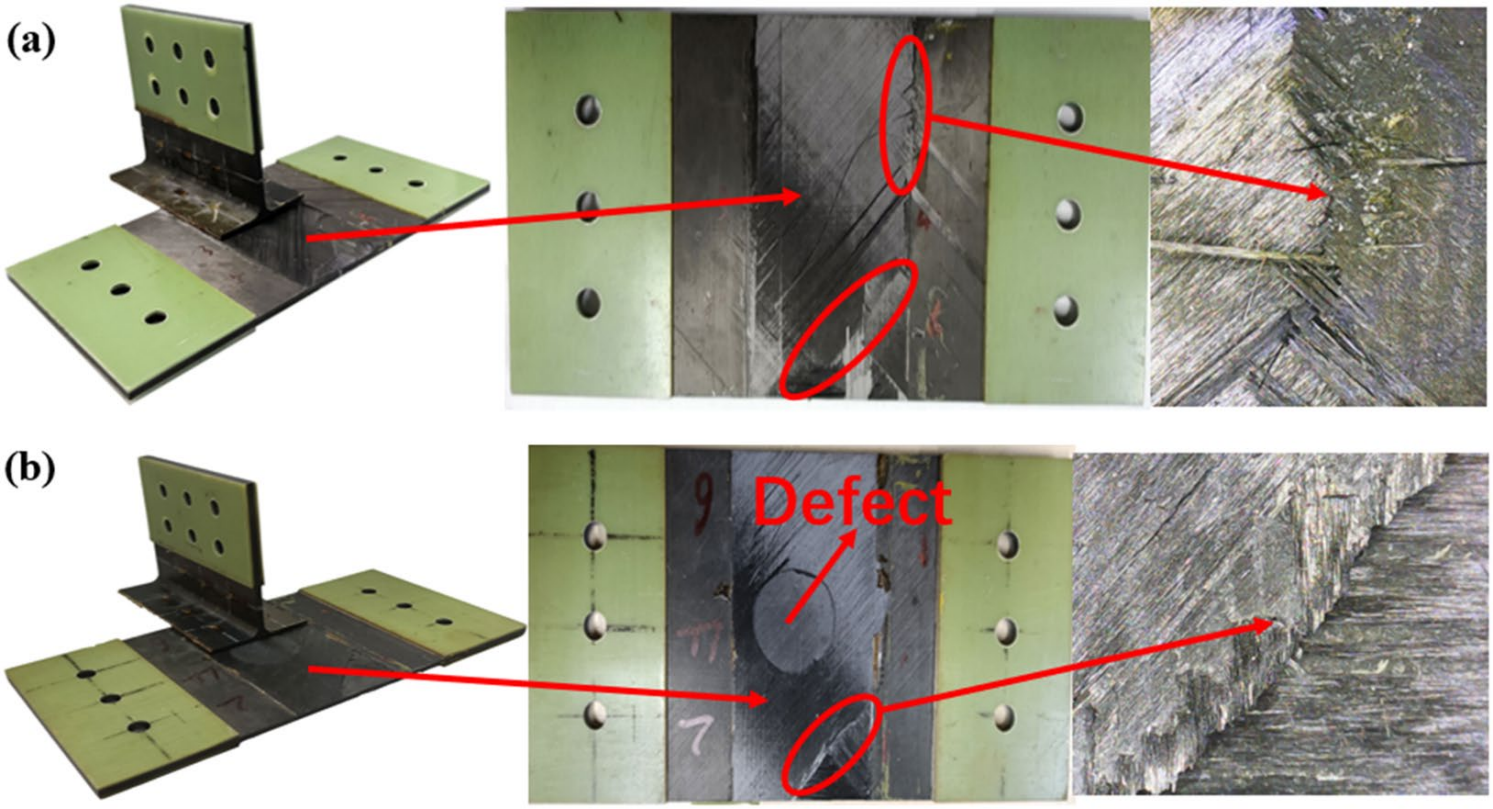
The results of the interface debonding test: (**a**) debonding appearance on side bending without defect; (**b**) debonding appearance on side bending with defect.

**Figure 11:**
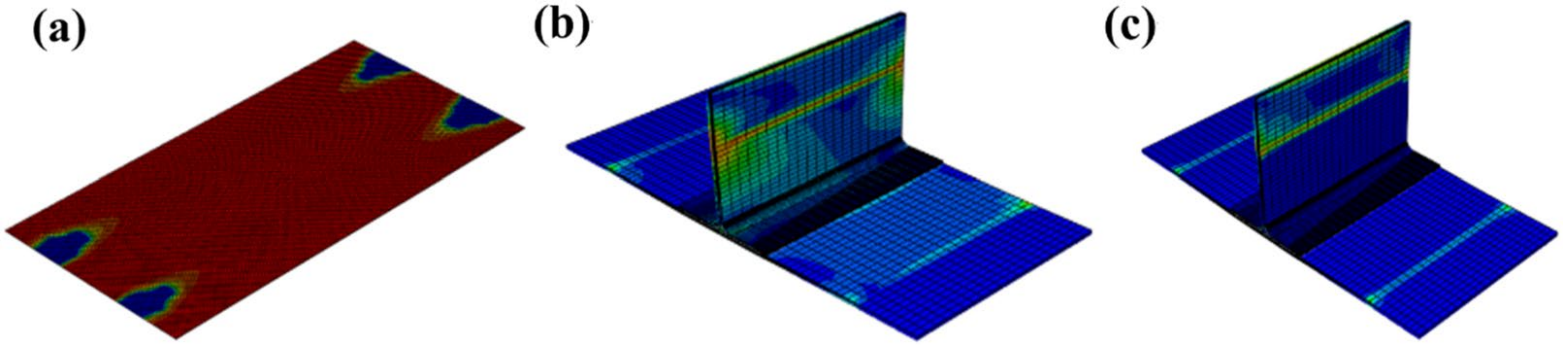
Shearing of T-stiffened panel without prefabrication defects: (**a**) interface failure; (**b**) matrix failure; (**c**) laminate failure (ABAQUS 6.14, https://www.3ds.com/products-services/simulia/products/abaqus)^43^.

**Figure 12:**
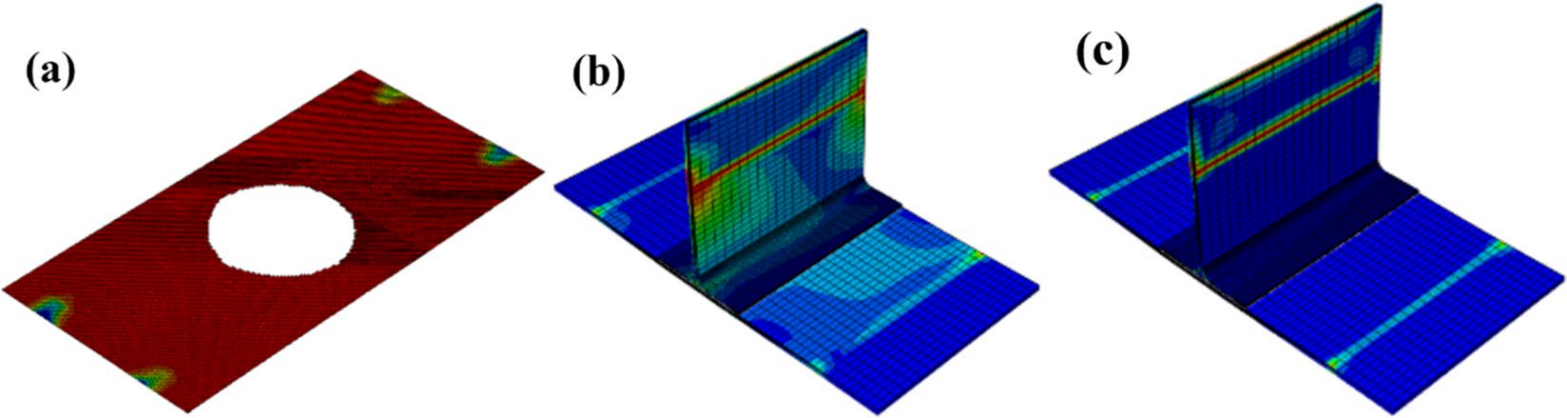
Shear of T-stiffened panel with prefabricated defects: (**a**) interface failure; (**b**) matrix failure; (**c**) laminate failure (ABAQUS 6.14, https://www.3ds.com/products-services/simulia/products/abaqus)^43^.

From the experimental results in Fig. 10, it can be seen that the T-type ribs are completely separated from the skin, and the intact T-shear has irregular fiber fractures at the connection interface, and cracks in the glue layer, large-area delamination, and a small amount of fiber is pulled out (Fig. 10a). The failure mode of the structure under shear load is type II and type III mixed failure. The stress field at the crack tip decreases slowly under shear load, so the damaged area at the crack tip of type II and type III is wider than that of type I in lateral bending. The defected T-stiffened panels expand regularly along both sides of the damaged area, and the fiber fractures are relatively neat. This is because the area containing the defected layer is given the initial damage and the damage evolves along a certain path. The fracture of the fiber along the shear direction shows that the fiber has a certain hindering effect on the shear failure of the interface adhesive. According to the analysis results of damage models in Figs. 11a and 12a, there are two paths for damage propagation of T-type structures without defects: one extends from both sides perpendicular to the loading direction to the middle; the second is from the joint center along the direction of the load to both ends of the expansion. The defected T-stiffened panel extends from the circular damage location in a direction parallel to the load. It can be seen from Figs. 11b to c and 12b to c that the T-stiffened panel produces large matrix compression damage and delamination near the loading end due to the high-stress level near the loading end, whether with or without prefabricated defects, which is consistent with the experimental results.

### Research on the influence law of design parameters

#### Layer direction

The surface layer will affect the interface bonding performance of the structure^45^, thereby affecting the load-bearing capacity of the structure. The ratio of 90: ± 45:0 in the original ribs (ply 2, 3) in each layer direction is 1:9:2. For the T-stiffened pane28l with prefabricated defects, two optimized design schemes with a ratio of 90: ± 45:0 of 1:2:1 and a ratio of 90: ± 45:0 of 2:3:1 are proposed, as shown in Table 7.

**Table 7:** Laying direction and proportion of ply.

Ply	
	**Plan 1 (1:2:1)**
2	[45/90/0/45/0/− 45/0/90/− 45/90/45/− 45]
3	[− 45/45/90/− 45/90/0/− 45/0/45/0/90/45]
	**Plan 2 (2:3:1)**
2	[45/90/0/90/45/− 45/0/45/− 45/90/45/90]
3	[90/45/90/− 45/45/0/− 45/45/90/0/90/45]

**Figure 13:**
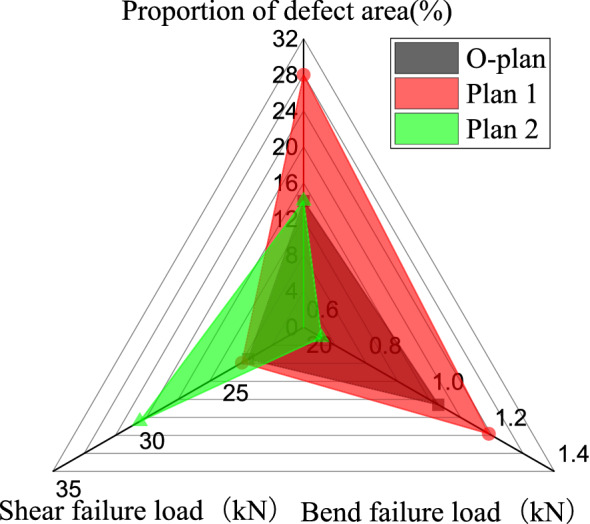
Finite element calculation results of various paving schemes.

For damaged T-stiffened bend, the finite element calculation results of the original plan(O-plan) and plan 1 and 2 are shown in Fig. 13 and Table 8. The finite element calculation of the original paving plan is 0.657 kN, and the failure load of plan 1 is 1.030 kN. The failure load is 1.191 kN, and the strength and stiffness of the two optimization plans are improved compared with the original plan. For T-shear with defects, the strength calculated by the finite element method of the original layup plan is 5.959 MPa, the strength of plan 1 is 4.663 MPa, and the strength of plan 2 is 4.736 MPa. Compared with the original plan, the strength and stiffness of the two optimization plans are lower. Side bending and shearing loads belong to two different types of loads. It can be seen from Fig. 13 that the increase in the side bending capacity does not mean the corresponding increase in the shear capacity.

**Table 8:** Research on the influence law of design parameters about defective specimen.

	Lateral bending load (kN)	Shear strength (MPa)
**Laying direction and proportion of ply**		
O-Plan	0.657	5.959
Plan 1 (1:2:1)	1.030	4.663
Plan 2 (2:3:1)	1.191	4.736
**Fillet radius**		
Plan 1 (R = 6 mm)	0.851	6.02
Plan 2 (R = 8 mm)	0.984	6.101
**Rib width**		
Plan 1 (40/80)	0.686	5.31
Plan 2 (60/120)	1.073	6.139

Since T-type lateral bending damage is mainly located in the interface layer, finite element analysis found that the interface layer damage under the side bending load of the three plans is mainly on the side bearing the load, and the initial damage and damage evolution law are not much different from the original plan. Due to the change of the bonding surface layer, when the lay angle changes from 0° to − 45° to 90°, the area of the completely debonded interface gradually decreases from the edge to the center of the adhesive layer interface, and the left and right sides are asymmetrical.

#### Fillet radius

**Figure 14:**
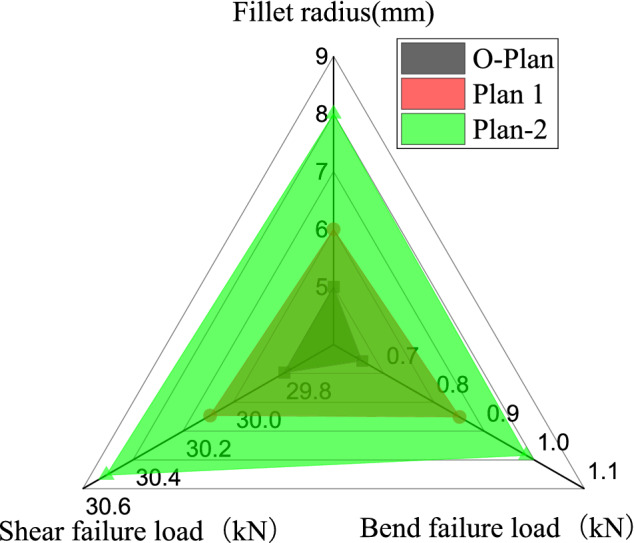
Finite element calculation results of the fillet radius scheme.

Aiming at the original T-stiffened panel with prefabricated defects and rounded corners R = 5 mm, two optimized design plans are proposed: Plan 1 (R = 6 mm) and Plan 2 (R = 8 mm). The finite element calculation results of the original Plan and Plan 1 and 2 are shown in Fig. 14 and Table 8. Under lateral bending load: the failure load calculated by the finite element method of the original plan is 0.657 kN, the failure load of plan 1 is 0.851 kN, and the ultimate load of plan 2 is 0.984 kN. Both plans 1 and 2 have higher carrying capacity than the original plan. Under shear load: the strength of plan 1 is 6.02 MPa, the strength of plan 2 is 6.101 MPa, and the optimized plan is also stronger than the original plan.

Under lateral bending load, as the fillet radius increases, the structural bearing capacity increases. Under the shear load, the damage of the T-stiffened panel expands from both sides to the center. Even if the fillet radius increases, the final skin, and ribs are still completely debonded, which is not much different from the failure form of the original plan. The results show that under the lateral bending load of the structure with defects, the structure with the fillet radius R = 8 mm has the highest strength, the structure with R = 6 mm is the second, and the original plan (R = 5 mm) is the lowest. Under the shear load, the structure with the defected structure has the highest strength with the fillet radius R = 8 mm, the structure with R = 5 mm is the second, and the plan with R = 6 mm is the lowest. As the fillet radius increases, the strength of the structure gradually increases. This is because the increase in the radius of the fillet can alleviate the stress concentration in the triangle area and also change the nature of load transfer.

#### Rib width

**Figure 15:**
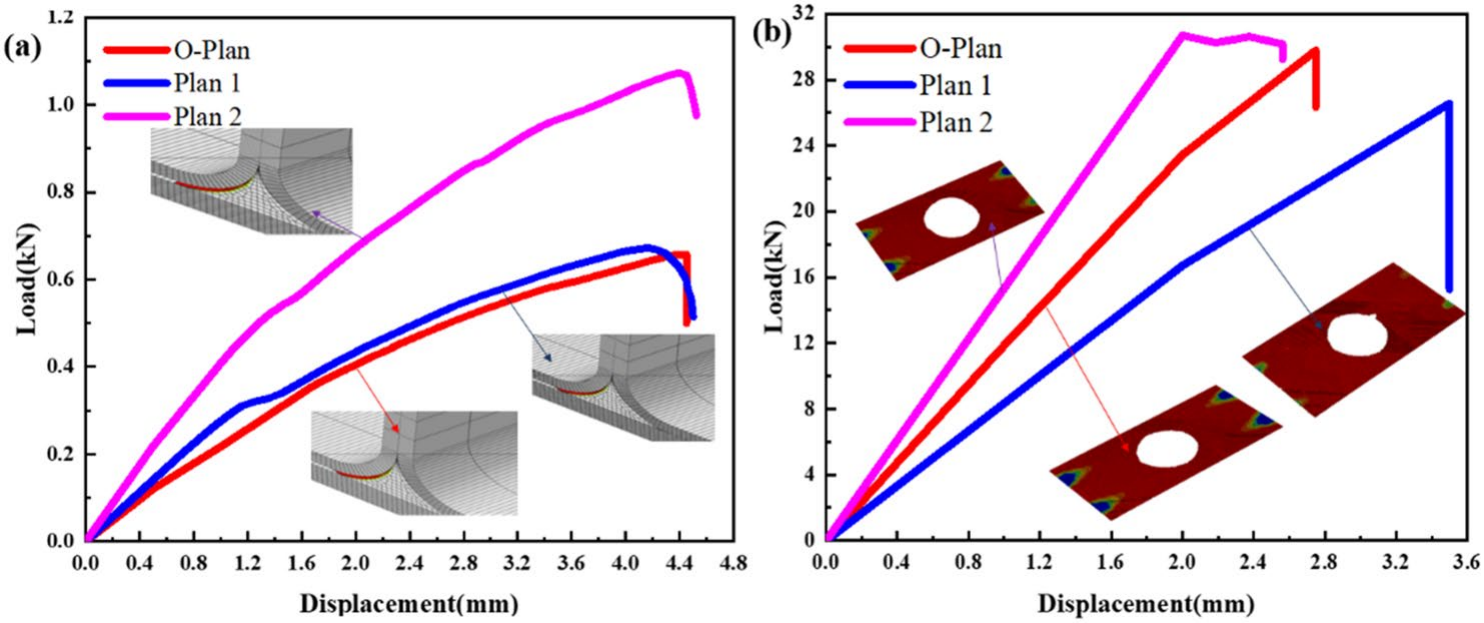
The simulation results: (**a**) comparison of finite element calculation results of various lateral bending plans; (**b**) comparison of finite element calculation results of various shearing plans (ABAQUS 6.14, https://www.3ds.com/products-services/simulia/products/abaqus)^43^.

For the original lateral bend and sheared rib width (L = 50 mm/100 mm) with prefabricated defects, two optimization plans (plan 1: L = 40/80 mm and 2: L = 60/120 mm) are proposed. The finite element results of the original plan and the optimized plan are shown in Fig. 15a,b and Table 8.

According to the finite element simulation, under the side bending load, the wider the rib width is, the more uniform the stress distribution of the bond interface will be. The triangular area is still the most initial damage location due to the influence of stress concentration, and the damage form of the bond interface has little difference from the original plan. The finite element analysis shows that the maximum bearing capacity of the structure with defects is 1.073 kN when the width of the ribs (L = 60 mm) is under bending load, and the bearing capacity of the original plan (L = 50 mm) is similar to that of the width of the ribs (L = 40 mm). Under shear load, the maximum strength of the structure with defects is 6.139 MPa with the width of the ribs (L = 120 mm), followed by the original plan (L = 100 mm), and the lowest strength is 5.31 MPa with the width of the ribs (L = 80 mm). Although the width of the 2 ribs in the optimized plan is 2 times that of the original plan, the strength is similar to that of the original scheme.

## Conclusion

In this paper, by adopting a combination of experiment and numerical simulation, we studied the high-strength and high-modulus composite T-stiffened panel, with intact and defected structure, its structural mechanical properties, and damage evolution law under lateral bending and shear loading. The comparative study shows:

1. The interface damage will reduce the side bending and shear bearing capacity of the structure. Compared with the shear load, the interface damage has a greater impact on the structural performance under the lateral bending load.
2. Under the lateral bending load, the damage mainly occurs at the bonding interface between the skin and the ribs on the loaded side and the corners of the L-type ribs. When there is a defect, the crack first extends from the position of the defects to both ends of the interface layer, and then the crack in the fillet area extends upward along the L-type bars on the one hand, and downward to the bonding interface between the skin and L-type ribs on the other hand. Partial shear damage occurred on the fiber and matrix on one side of the load, and some compression damage occurred on the fiber and matrix on the other side. Part of the matrix tensile damage was also caused by the stress concentration at the corners of the L-type ribs.
3. The structural damage under shear load spreads from both sides to the center. When prefabricated defects are included, the damage starts from the defect position in the center of the interface and expands outward, and at the same time, the two sides also expand to the center until it fails completely. Due to the high-stress level near the loading end, the T-stiffened panel produced larger matrix compression damage and laminate delamination near the loading end.
4. In the structural design parameters, as the fillet radius increases, the load-bearing capacity of the T-stiffened panel also increases. In addition, the width of the bonding surface layer and the ribs also have a certain effect on the interface debonding.
